# Study of the Mechanical Properties of Highly Efficient Heat Exchange Tubes

**DOI:** 10.3390/ma13020382

**Published:** 2020-01-14

**Authors:** Caifu Qian, Zhiwei Wu, Shihang Wen, Shaoping Gao, Guomin Qin

**Affiliations:** 1 College of Mechanical and Electrical Engineering, Beijing University of Chemical Technology, Beijing 100029, China; zwzhiweiwu@163.com (Z.W.); wenshihang@126.com (S.W.); 2 Daqing Petro-Chemical Machinery Factory, Daqing 163714, China; gaosp1-ds@petrochina.com.cn (S.G.); qinguomin911@163.com (G.Q.)

**Keywords:** highly efficient heat exchange tubes, stress analysis, axial stiffness, fatigue strength, numerical simulation

## Abstract

Highly efficient heat exchange tubes are special tube shapes that are widely used in heat exchangers to enhance heat transfer. In this study, experimental measurements and numerical simulations were carried out on two types of highly efficient heat exchange tubes, namely, spirally grooved tubes and converging–diverging tubes, to investigate changes in their mechanical properties after rolling from smooth tubes. It was found that, unlike the smooth tubes, all axial, circumferential, and radial stresses exist at the two types of tubes under axial loading, and the maximum axial stress is much larger than that at the smooth tubes. Compared to the smooth tubes, the yield strength and ultimate strength of the highly efficient heat exchange tubes increase while the axial elastic stiffness decreases. Although the capability of resisting fatigue fracture of the highly efficient heat exchange tubes is less than that of smooth tubes, they still meet the requirements of the heat exchanger under fatigue loading. Axial stress concentration factors and stiffness equivalent factors for the highly efficient heat exchange tubes are regressed as a function of the structural parameters for engineering applications.

## 1. Introduction

With the rapid development of industry, a lot of methods to enhance heat transfer have been developed, among which highly efficient heat exchange (HEHE) tubes are most commonly used in engineering. A lot of research has been performed in the literature on the heat transfer performance of heat exchangers with HEHE tubes [1,2,3,4,5]. Kareem et al. [6] presented an extensive review of numerical and experimental studies on the heat transfer enhancement of corrugated tubes, which cover laminar and turbulent flow. In order to improve the heat and mass transfer performance of falling film and develop a new type of falling film evaporator, Huang et al. [7] studied the heat and mass transfer characteristics of falling film evaporation and sensible heat in four different sizes of converging–diverging tubes. Rashid et al. [8] presented a novel model of a parabolic trough-based solar thermal and natural gas hybrid power plant. Due to the intermittence of solar energy, the model can make use of natural gas combustion to supplement steam when cloud cover and solar intensity are reduced, which can improve the reliability and efficiency of solar power generation. It is worth noting that the system uses a shell and tube heat exchanger to extract more heat from the heat transfer fluid before returning to the parabolic trough. In this case, HEHE tubes are very effective in improving energy efficiency.

Due to the irregular shape and rolling from the smooth tubes, the load-carrying capability of HEHE tubes may be quite different from that of smooth tubes, of which sufficient attention should be paid in order to ensure reliable operation of heat exchangers constructed with HEHE tubes. Eyvazian et al. [9,10] studied the effect of crushing parameters on an aluminum corrugated tube and the compression response under transverse quasi-static loads by corrugations of different geometries through experiments and numerical simulations. The results showed that by changing the shape of the corrugations, the stress distribution pattern changed significantly. Wang et al. [11] used the finite element method to simulate and analyze corrugated tubes in the heat exchanger. An analysis method for the stability of the corrugated tubes under internal and external pressure was proposed, and the calculation formula of single wave stiffness was obtained. Singace et al. [12] conducted an experimental study on the energy absorption characteristics of corrugations. The experimental results show that the corrugations are an ideal controllable energy-absorbing element. Song et al. [13] analyzed the stiffness and strength of cylindrical rods and tubes from material mechanics. It was found that when the masses of the two were the same, the cross-section stiffness of the circular tube was three times that of the cylindrical rod. Using the basic theory of elastoplasticity, the yield stress of a liquid-filled cylindrical tube was studied. Due to the incompressibility of the liquid and the strain hardening effect of the material, the yield stress of the liquid-filled tube increased and the bending strength increased. Under the dynamic load, compared to the hollow tube, the impact resistance of the liquid-filled tube was also raised due to elastic recovery. Li et al. [14] studied the influence of heat transfer tube stiffness and tube sheet thickness on the thermal stress of tubes and tube sheets. It was found that the use of corrugations to reduce the stiffness of the tube can reduce the axial thermal stress at the tube, thus reducing the possibility of failure of the connection strength between the tube and the tube sheet. Qian et al. [15] conducted numerical and experimental research on the axial stiffness of the corrugated tubes. Based on the analysis of axial load-displacement, the stiffness weakening factor *K_f_* of the corrugated tubes was proposed and formulated. Yang et al. [16] analyzed the axial stiffness of the twisted tube by finite element and experimental methods. The results showed that the increase in the twist ratio or decrease in the lead will lead to the decrease in axial stiffness, and a formula for reducing the stiffness of the twisted tube was proposed. Shen et al. [17] analyzed the leakage of titanium tubes in condensers of nuclear power plants, including analysis of the chemical composition of the alloy, mechanical properties, metallographic structure, and micro-morphology. The results showed that the leakage of the titanium tube is mainly caused by fatigue failure. Fatigue tests were carried out in air and steam environments, and the fatigue resistance of the titanium tube in the steam environment was significantly reduced. Vetriselvan et al. [18] established an experimental device to simulate thermal fatigue on the inside diameter side of the tube. Based on the decoupling analysis of thermal and inelastic stresses, finite element analysis was performed to calculate the total plastic strain range of the boiler tube. Finally, the number of cycles of crack initiation was obtained through experiments, and the number of cycles of crack initiation was calculated using the modified Coffin–Manson relation. It provides a reliable basis for the fatigue failure analysis of ^9^Cr^1^Mo steel tubes for boilers. Sajuri et al. [19] characterized the metallurgical, mechanical, and fatigue properties of copper–phosphorus alloys and aluminum–copper bimetallic tubes by metallographic analysis and tensile, bending, and fatigue tests. The results showed that a fragile Al–Cu intermetallic compound was found in the transition layer between Al and Cu. The bending performance of the tube was affected by the volume fraction of Cu in the material. The fatigue strength of the aluminum–copper bimetal tube was reduced by nearly 55% compared to the copper alloy tube. Leber et al. [20] studied the microstructure changes of isothermal low-cycle-fatigue samples made from industrial steel tube processing. The volume fraction of martensite determined by the optimized magnetic nondestructive test method was generally small, but it was large near the crack tip with high plastic strain, and martensite was found at the intersection of the slip zone. Hsu [21] studied the fatigue fracture of the industrial pure titanium tube in the shell and tube heat exchanger at low temperature. As industrial titanium tubes are subjected to vibration and compressive axial loads caused by flow during use, the combined stress causes many intergranular cracks in the circumferential direction. Fracture analysis showed that the fracture was caused by high cycle fatigue. 

As HEHE tubes, spirally grooved (or SG) tubes and converging–diverging (or CD) tubes are widely used in heat exchangers. However, a literature review found that with the considerable research addressing the heat exchange enhancement properties of the HEHE tubes and some on the load-carrying capability of HEHE tubes, there are few studies on the changes in their mechanical properties after rolling from smooth tubes. In this paper, the mechanical properties of the SG tubes and CD tubes are studied and compared to those of smooth tubes. The purpose is to ensure the reliable use of HEHE tubes in heat exchangers.

## 2. Structure of the Studied HEHE Tubes 

**Figure 1:**
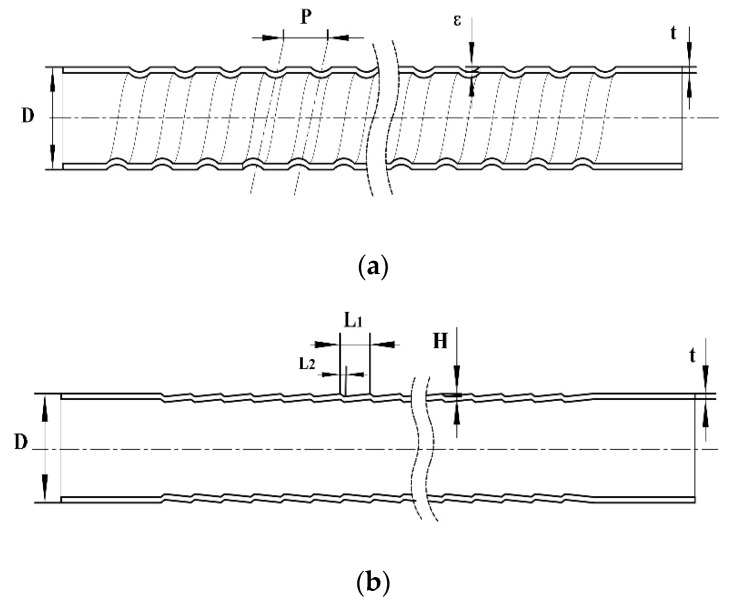
Schematic structures of two highly efficient heat exchange (HEHE) tubes: (**a**) Spirally grooved (SG) tube, (**b**) Converging–diverging (CD) tube.

In this paper, SG tubes and CD tubes are studied numerically and experimentally. Figure 1a illustrates the structure of the SG tubes, where *D* is the nominal outer diameter of the tube, which is equal to the outer diameter of the smooth tube before rolling, *P* is the pitch of the tube, *ε* is the depth of the spiral groove, and *t* is the thickness of the tube. Figure 1b illustrates the structure of the CD tubes, where *D* is the nominal outer diameter of the tube, which is also equal to the outer diameter of the smooth tube before rolling, *L*_1_ is the length of a period section, *L*_2_ is the length of the tapering section, which is about 1/8 *L*_1_, and *H* is the rib height. The materials of the above two HEHE tubes are carbon steel No. 10 or austenitic stainless steel S30408.

In engineering applications, heat exchange tubes generally undertake pressures inside, as well as outside, the tubes, and also axial loading. As the tubes are small in cross-section but long in length, axial loading is critical for the reliable use of the tubes. Therefore, in this paper, mechanical properties of the SG tubes and CD tubes under axial loads are investigated experimentally and numerically to see if the load-carrying capability of these HEHE tubes changes after rolling from smooth tubes.

## 3. Experimental Measurements 

The purpose of the experimental study is to investigate changes in the mechanical properties of the HEHE tubes after rolling from smooth tubes.

### 3.1. Axial Strength and Stiffness

**Figure 2:**
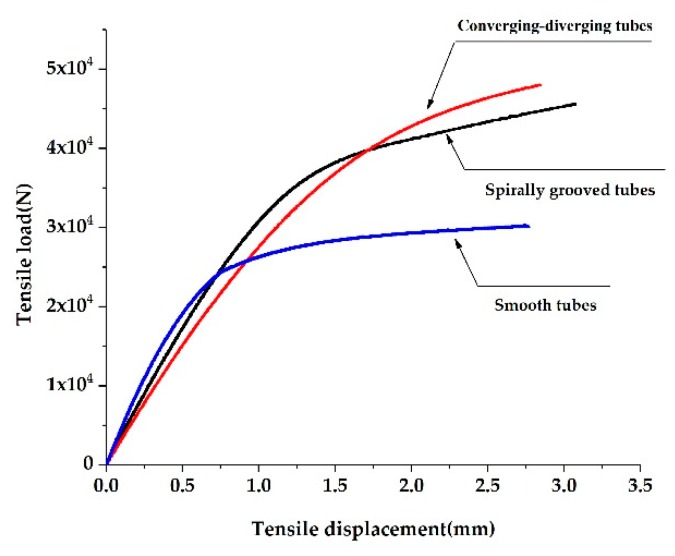
Comparison of experimental load-deformation curves of the two HEHE tubes and a smooth tube.

For a tube with a given length, axial stiffness refers to the axial deformation of the tube under unit axial tensile or compressive load. In this study, the axial stiffness of two HEHE tubes is tested on an Instron-8800 electro-hydraulic servo universal material testing machine (Boston, MA, USA). The measuring range of the extensometer is 50 mm and the measuring accuracy is 1 micron. Figure 2 compares the tensile load and displacement curves of a SG tube, CD tube, and smooth tube with their diameter of 19 mm and thickness of 2 mm. Test results for tubes with other sizes are similar to Figure 2.

From Figure 2, it is found that the smooth tube is the first to present a yield trend under the tensile loading. As a result of straining–strengthening during the rolling process, the yield strength and ultimate strength of the SG tube and CD tube are enhanced. In the elastic stage, the slope of the straight line represents the stiffness of the tubes. Clearly, as the cross-section changes periodically, the axial elastic stiffness of the SG tube and CD tube is lower than that of the smooth tube.

In order to deeply investigate the stiffness of HEHE tubes, the ratio of the stiffness of HEHE tubes to that of smooth tubes under the same nominal outer diameter and thickness is defined as the stiffness equivalent factor $K_{f}$.

(1) $$K_{f}=\frac{K_{1}}{K_{2}},$$

where $K_{1}$ is the axial elastic stiffness of the HEHE tubes and $K_{2}$ is the axial elastic stiffness of the smooth tubes. Obviously, $K_{f}$ must be related to the structural parameters of HEHE tubes. The closer $K_{f}$ is to 1, the closer the axial elastic stiffness of the HEHE tubes is to that of the smooth tubes with the same nominal outer diameter and thickness.

Several groups of tensile tests were carried out to measure $K_{f}$ of the SG tubes and CD tubes. The experimental results are listed in Table 1 and Table 2 respectively for the two HEHE tubes with different sizes. 

### 3.2. Fatigue Strength

In engineering, some heat exchangers with highly efficient heat exchange tubes undergo changing pressures and/or temperatures. Therefore, it is necessary to know the change in fatigue-resistant properties of the HEHE tubes after rolling from the smooth tubes and forming structural discontinuities. In this part, the fatigue tests were carried out on the SG tubes and CD tubes. The materials of the tested tubes were carbon steel No. 10 and austenitic stainless steel S30408 with their yield strength being 205 and 210 MPa, respectively. The maximum load $F_{max}$ for the fatigue tests was determined as follows [22]:

(2) $$\sigma_{max}=\frac{\sigma_{s}}{n},$$

(3) $$F_{max}=\sigma_{max}•S,$$

where $\sigma_{s}$ is the yield strength of the material, *n* is the safety factor, and *S* is the cross-sectional area of the base tube.

**Figure 3:**
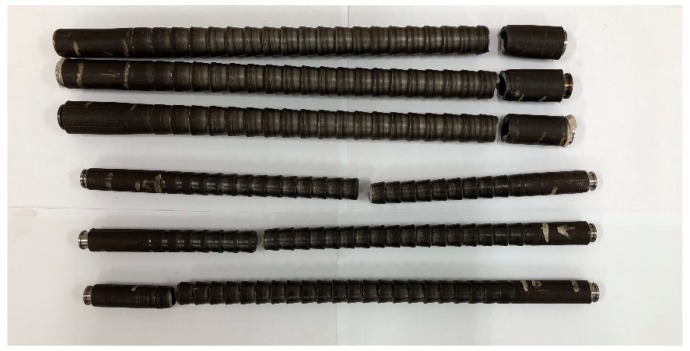
Some fractured CD tubes.

In engineering applications, the axial stress of heat exchanger tubes is usually not allowed to exceed the allowable stress of the material for which the safety factor n corresponding to the yield strength $\sigma_{s}$ is 1.5. For the fatigue tests in this study, $\sigma_{max}$ was also determined by taking *n* = 1.5. The tests were carried out under tensile loading with the load ratio *R* (the minimum load to the maximum load) being 0.01. The results of fatigue tests in each group are listed in Table 3 and Table 4 respectively for SG tubes and CD tube. Figure 3 shows some fractured fatigue test specimens. Smooth tubes with the same materials were also tested under the maximum loading with *n* = 1.5 in Equation (2), and no tubes were fractured within 1,000,000 cycles.

From Table 3 and Table 4, it is seen that for the SG tubes, when the maximum load is determined by Equations (2) and (3) with *n* = 1.5, all specimens underwent 1,000,000 cycles without breaking. For CD tubes, however, as listed in Table 4, most tested specimens were broken with a cycle less than 1,000,000 but more than 100,000. Thus, it seems that SG tubes are more capable of resisting fatigue fracture than CD tubes. This may be owed to the fact that CD tubes more sharply change in structural profile, and it is easier for them to initiate fatigue cracks than SG tubes after rolling.

As mentioned above, in engineering applications, the axial stress of heat exchange tubes is usually controlled to be less than the allowable stress, which is obtained by Equation (2) with *n* = 1.5. In addition, most heat exchangers, if fatigue-loaded, undergo low cycle fatigue or, in other words, the working cycle is less than 100,000. Thus, it is reached that although the capability of resisting fatigue fracture of the SG tubes and CD tubes may be less than that of smooth tubes, they still meet the requirements of engineering applications.

It turns out that the fracture of tested specimens was all initiated from the trough of the outer surface of the CD tubes, which is clearly due to the stress concentrations in these places. The next section will numerically study the stress distributions under the axial loading with more attention on the stress concentrations.

## 4. Numerical Simulations

### 4.1. Numerical Models

**Figure 4:**
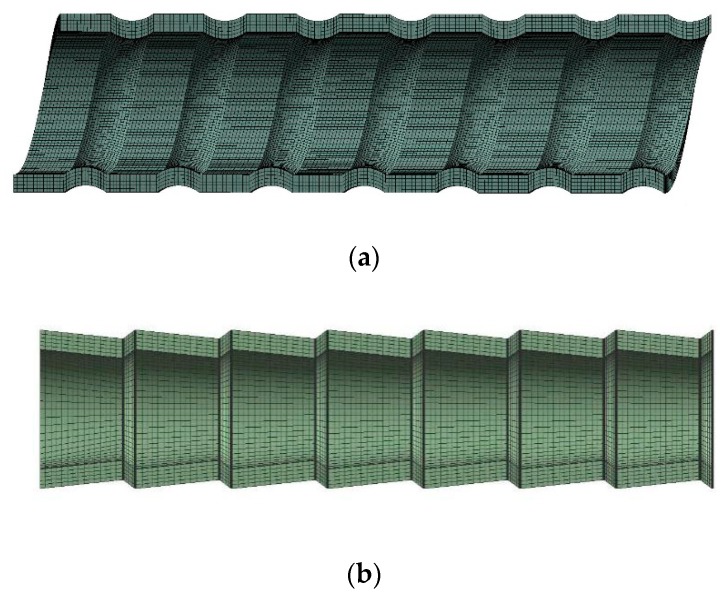
Mesh models of two HEHE tubes: (**a**) SG tube, (**b**) CD tube.

In this study, hexahedral elements are used to establish the finite element mesh models of the tubes. In order to ensure the accuracy of the numerical calculation, mesh independence is verified. The results are shown in Table 5 and Table 6 respectively for the SG and CD tube. It is seen that for the three listed mesh methods, their differences in stress intensity are very small. Based on the results, the mesh models with 2.8 million meshes for the SG tube and 1.2 million meshes for the CD tube are employed in the following analysis. In addition, in this study, the axial tensile load is applied on one end of the HEHE tubes, while the other end is fixed. To eliminate the local effects of the ends, the analysis results in the middle section of the tubes are considered. Figure 4 shows the mesh models of the middle segment of the SG tube and CD tube.

### 4.2. Stress Distributions 

As mentioned before, axial loading is critical for HEHE tubes in engineering applications. Thus, in this section, stress distributions are investigated at the SG tubes and CD tubes under the axial loading and will be compared to those at the smooth tubes with the same nominal size and under the same loading. If only for comparison, the magnitude of the axial loading is not meaningful. In this study, the axial loading *F* is set to be 5000 N under which the deformation of the tube is elastic.

**Figure 5:**
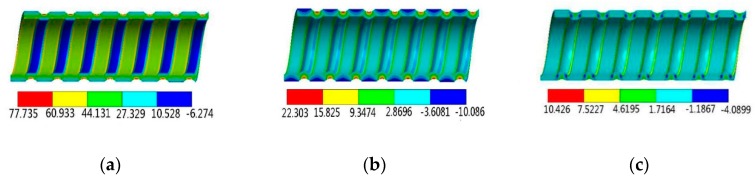
Three-dimensional stress distributions at the SG tubes: (**a**) Axial stress, (**b**) Circumferential stress, (**c**) Radial stress.

**Figure 6:**
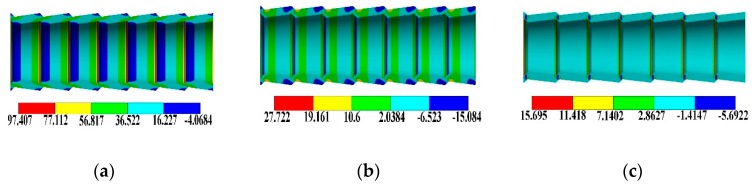
Three-dimensional stress distributions at the CD tubes: (**a**) Axial stress, (**b**) Circumferential stress, (**c**) Radial stress.

Figure 5 and Figure 6 show the three-dimensional stress distributions at the SG tubes and CD tubes, respectively. It is seen that under the axial loading, axial stress, circumferential stress, and radial stress all exist at the tubes, which is totally different from the smooth tubes where only axial stress exists under the axial loading. Obviously, the circumferential stress and radial stress are caused by the bending moments, which are induced by the axial loading acting on the periodically changing cross-section of the HEHE tubes. 

**Figure 7:**
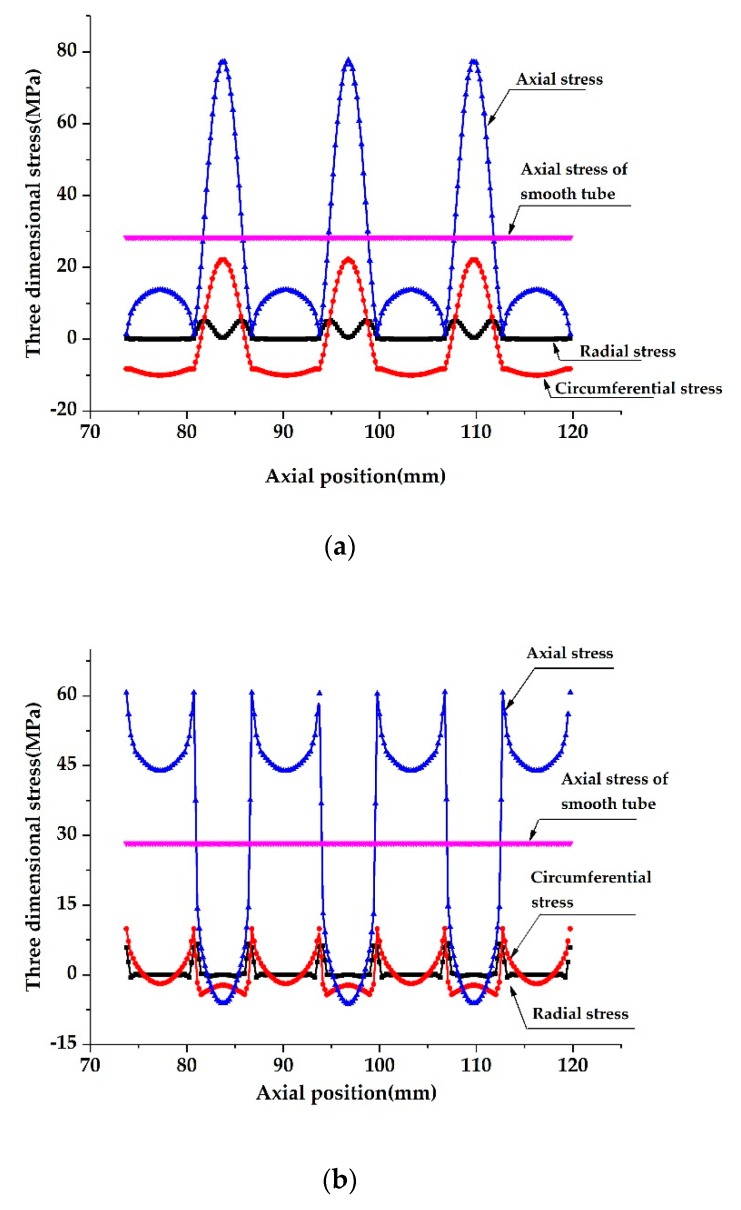
Stress distributions along tube length on inner and outer surfaces of the SG tubes: (**a**) On the outer surface, (**b**) On the inner surface. (*D* = 25 mm, *P* = 13 mm, *t* = 2.5 mm, *ε* = 1 mm, *F* = 5000 N).

**Figure 8:**
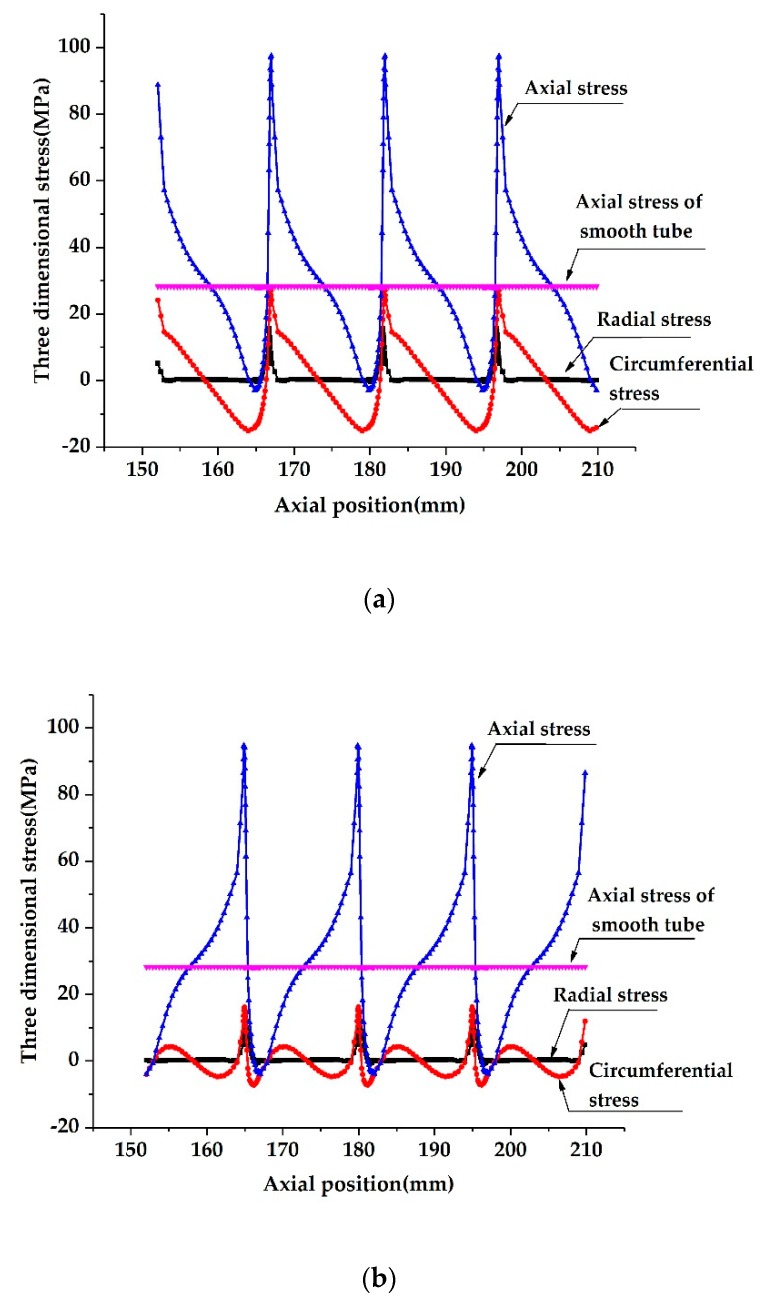
Stress distributions along tube length on inner and outer surfaces of the CD tubes: (**a**) On the outer surface, (**b**) On the outer surface. (*D* = 25 mm, *L*_1_ = 15 mm, *t* = 2.5 mm, *H* = 1.25 mm, *F* = 5000 N).

Figure 7 and Figure 8 show the stress distributions on the outer and inner surfaces of the SG tube and CD tubes along the tube length. Due to the periodic expansion and contraction of the cross-section of HEHE tubes, the stress distribution presents a complex cyclical trend. It is seen that under the action of axial tensile load, the axial stresses on the inner and outer surfaces are the most significant and change rapidly along the tube length. Specifically, at the bottom of the groove on the outer surface of the SG tube, the axial stress is the largest, which is about 2.8 times larger than that of the smooth tube with the same nominal size under the same loading condition. On the inner surface of the SG tube, the maximum axial stress appears near the two sides of the spiral groove, which is about 2.2 times larger than that at the smooth tube. The maximum value of the axial stress on the inner surface of the CD tube is about 3.3 times larger than that at the smooth tube and appears at the top of the tensile section, and the maximum value on the outer surface is 3.5 times larger and appears at the bottom of the tapered section.

It should be noted that for a structure made from a ductile material, peak stress may not affect the static load-carrying capability of the structure, but cracks could be initiated in the places with peak stress and, thus, decrease the fatigue strength of the structure. In this study, although the SG tube and CD tubes may have stronger static load-carrying capabilities as the yield strength and ultimate strength are enhanced because of the work hardening, their fatigue strength is reduced as cracks could be easily initiated compared to the smooth tubes. 

In addition, although the axial stress at the above two HEHE tubes changes significantly along thickness of the tube, the average axial stress is the same as that at the smooth tube if the thickness of the HEHE tubes does not change after rolling. In addition, the radial stress in Figure 7 and Figure 8 should be understood as the stress close to the surfaces, because the radial stress at the outer or inner surfaces must be zero.

As mentioned above, if the HEHE tubes are fatigue-loaded, the maximum stress or peak stress at the tubes is critical because the peak stress could initiate a fatigue crack and lead to fracture of the tubes. Therefore, in this study, the maximum axial stress at the SG tubes and CD tubes are evaluated by introducing a so-called axial stress concentration factor $R_{z}$, which is defined as the ratio of the maximum axial stress at the HEHE tubes over the axial stress at the smooth tubes with the same nominal size under the same axial load. 

For the SG tubes, if we define $R_{Z}^{S}$ as the axial stress concentration factor, it turns out that $R_{Z}^{S}$ is mainly affected by the tube structural parameters *t* and *ε*. By defining dimensionless variables *ε/D* and *t/D* and using the multivariate linear regression method based on enough numerical results regarding the influences of these variables on $R_{Z}^{S}$, the axial stress concentration factor $R_{Z}^{S}$ can be regressed into the following formula:

(4) $$\begin{array}{ll} R_{Z}^{S}= & -2035.93\frac{\varepsilon }{D}\cdot {\left(\frac{t}{D}\right)}^{2}+121.598\frac{t}{D}\cdot \frac{\varepsilon }{D}+85.5995\frac{\varepsilon }{D}\\ & +157.108{\left(\frac{t}{D}\right)}^{2}-29.1994\frac{t}{D}+1.1037 \end{array}.$$

Equation (4) is applicable for D = 19 or 25 mm, 1.5 ≤ t ≤ 3.5 mm, 0.8 ≤ *ε* ≤ 1.2 mm, and P = 13 mm. Within these ranges, $R_{Z}^{S}$ is about 2.2–2.8.

Similarly, for the CD tubes, we can define $R_{Z}^{C}$ as the axial stress concentration factor. It is found that $R_{Z}^{C}$ is mainly affected by structural parameters *t* and *H*, By defining dimensionless variables *t/D* and *H/D* and investigating their effects on $R_{Z}^{C}$, a regression formula for $R_{Z}^{C}$ can be obtained based on sufficient numerical simulations:

(5) $$\begin{array}{ll} R_{Z}^{C}= & 3681.51\frac{H}{D}\cdot {\left(\frac{t}{D}\right)}^{2}-1358.18\frac{t}{D}\cdot \frac{H}{D}+155.397\frac{H}{D}\\ & -78.568{\left(\frac{t}{D}\right)}^{2}+26.338\frac{t}{D}-1.207 \end{array}.$$

The applicable scope of Equation (5) is as follows:

(1) D = 19 mm, L1 = 12 mm, 2.00 ≤ t ≤ 3.00 mm, 0.8 ≤ H ≤ 1.2 mm;
(2) D = 25 mm, L1 = 15 mm, 2.00 ≤ t ≤ 3.00 mm, 1.05 ≤ H ≤ 1.45 mm.

### 4.3. Axial Stiffness 

As found by experiments in Section 3.1, compared to the smooth tubes, the axial stiffness of two HEHE tubes decreases to some extent and, in this study, a so-called stiffness equivalent factor $K_{f}$ is defined there by Equation (1). In this section, numerical simulations are performed to deeply investigate $K_{f}$ and its influence factors.

Like the stress concentration factor $R_{z}$, the stiffness equivalent factor $K_{f}$ is also a function of the tube structural parameters. Specifically, for the SG tubes, the stiffness equivalent factor $K_{f}^{S}$ is mainly affected by the pitch *P*, depth *ε*, and thickness *t*. A lot of numerical calculations were carried out to find the relations between $K_{f}^{S}$ and *P*, and *ε* and *t*. By defining dimensionless variables *P/D*, *ε/D*, and *t/D* and using the multi-regression analysis method based on sufficient numerical results, $K_{f}^{S}$ can be regressed into the function of *P/D*, *ε/D*, and *t/D* as follows:

(6) $$\begin{array}{ll} K_{f}^{S}= & 56.0551\frac{\varepsilon }{D}\cdot \frac{t}{D}\cdot \frac{p}{D}-1.0540\frac{\varepsilon }{D}\cdot \frac{p}{D}-5.8063\frac{t}{D}\cdot \frac{p}{D}-12.7332\frac{\varepsilon }{D}\cdot \frac{t}{D}\\ & -9.8077\frac{\varepsilon }{D}+4.7160\frac{t}{D}+0.6357\frac{p}{D}+0.5841 \end{array}.$$

Equation (6) is applicable for *D* = 19 or 25 mm, 1.5 ≤ *t* ≤ 3.5 mm, 0.8 ≤ *ε* ≤ 1.2 mm, and 9 ≤ *P* ≤ 17 mm.

It turns out that for the CD tubes, the stiffness equivalent factor $K_{f}^{C}$ is mainly influenced by the structural parameters *H, t*, and *L*_1_. Similarly, by defining dimensionless variables *H/D, t/D*, and *L_1_/D* and using the multi-regression analysis method based on sufficient simulations, $K_{f}^{C}$ can be regressed as follows:

(7) $$\begin{array}{ll} K_{f}^{C}= & -95.71\frac{H}{D}\cdot \frac{t}{D}\cdot \frac{L_{1}}{D}+15.171\frac{H}{D}\cdot \frac{L_{1}}{D}+1.365\frac{t}{D}\cdot \frac{L_{1}}{D}+96.997\frac{H}{D}\cdot \frac{t}{D}\\ & -19.126\frac{H}{D}-1.350\frac{t}{D}-0.187\frac{L_{1}}{D}+1.229 \end{array}.$$

The scope of application of Equation (7) is as follows:

(1) D = 19 mm, 2.00 ≤ *t* ≤ 3.00 mm, 0.8 ≤ *H* ≤ 1.2 mm,10 ≤ *L*_1_ ≤ 14 mm.
(2) D = 25 mm, 2.00 ≤ *t* ≤ 3.00 mm, 1.05 ≤ *H* ≤ 1.45 mm,13 ≤ *L*_1_ ≤ 17 mm.

Equations (6) and (7) were verified by experimental measurements. Table 7 and Table 8 list the results respectively for SG tubes and CD tubes, where $K_{f}^{E}$ is the stiffness equivalent factors obtained by experiments and $K_{f}^{S}$ and $K_{f}^{C}$ are the stiffness equivalent factors respectively obtained by Equations (6) or (7), where the error is defined as ($K_{f}^{S}$ or $K_{f}^{C}$–$K_{f}^{E}$)/$K_{f}^{E}$. It is seen that the errors are less than 10%, implying that the accuracy of Equations (6) and (7) is acceptable in engineering. There could be several sources for the error, and the following two may be involved: (1) Error caused by deviation in the structure and size of the actual tubes from the ideal modelled tubes; (2) error caused by the deformation measurements of the extensometer, which is hard to tie to the non-smooth outer surfaces of the tubes during the test process. 

## 5. Conclusions

In this study, experimental measurements and numerical simulations were carried out to investigate changes in the mechanical properties of SG tubes and CD tubes after rolling from smooth tubes. The main conclusions are drawn as follows.

(1) Unlike the smooth tubes, as a result of the bending moments existing at the cross-section, all axial, circumferential, and radial stresses occur at the two HEHE tubes under the axial loading Among them, the maximum axial stress is much larger than that at the smooth tubes with the same nominal size under the same axial loading, implying that it is much easier to initiate cracks with the HEHE tubes than the smooth tubes.
(2) Experimental measurements show that compared to the smooth tubes, the yield strength and ultimate strength of the two HEHE tubes are enhanced as a result of work hardening while the axial elastic stiffness is decreased owing to the periodically changing cross-section. These results indicate that the HEHE tubes have a stronger load-carrying capability and easier deformation ability.
(3) Although the capability of resisting fatigue fracture of the two HEHE tubes is less than that of the smooth tubes, they still meet the requirements of engineering applications. Fatigue fracture is initiated at the trough of the changed surfaces where stress concentrations exist.
(4) Axial stress concentration factors $R_{Z}^{C}$ and $R_{Z}^{C}$ are defined for the two HEHE tubes and regressed as functions of the structural parameters, which could be applied in the strength design of these tubes in engineering.
(5) Stiffness equivalent factors $K_{f}^{S}$ and $K_{f}^{C}$ are defined for the two HEHE tubes, and formulas for $K_{f}^{S}$ and $K_{f}^{C}$ are also regressed as functions of the structural parameters for engineering application.

## Figures and Tables

**Table 1:** Experimental measurements of the stiffness equivalent factor of the SG tubes with different sizes.

No.	Tube Size				$K_{f}$
	*D*	*P*	*E*	*t*	
1	19	11.3	0.95	2	0.7583
2	19	11	0.85	2	0.7768
3	19	10.8	0.9	2	0.7625
4	25	12.5	0.9	3.1	0.8521
5	25	12.5	0.85	3	0.8724
6	25	12.5	1	3	0.8293

**Table 2:** Experimental measurements of the stiffness equivalent factor of the CD tubes with different sizes.

No.	Tube Size				$K_{f}$
	*D*	*L*_1_	*H*	*t*	
1	19	12	1.06	2.64	0.8481
2	19	12	1.08	2.64	0.8252
3	19	12	1.06	2.62	0.8367
4	25	15	1.40	3	0.7983
5	25	15	1.42	3	0.7896
6	25	15	1.36	3	0.8463

**Table 3:** Fatigue experimental results of the SG tubes with different sizes and materials.

No.	Tube Size				Safety Factors	Materials	Cycles	Results
	*D*	*P*	*ε*	*t*				
1	19	7.0	1.36	2.0	1.5	10	1,000,000	Unbroken
2	19	7.0	1.34	2.0	1.5	10	1,000,000	Unbroken
3	19	7.0	1.44	2.0	1.5	10	1,000,000	Unbroken
4	25	7.0	1.42	2.5	1.5	10	1,000,000	Unbroken
5	25	7.0	1.36	2.5	1.5	10	1,000,000	Unbroken
6	25	7.0	1.38	2.5	1.5	10	1,000,000	Unbroken
7	19	7.0	1.36	2	1.5	S30408	1,000,000	Unbroken
8	19	7.0	1.34	2	1.5	S30408	1,000,000	Unbroken
9	25	7.0	1.42	2	1.5	S30408	1,000,000	Unbroken
10	25	7.0	1.40	2	1.5	S30408	1,000,000	Unbroken

**Table 4:** Fatigue experimental results of the CD tubes with different sizes and materials.

No.	Tube Size				Safety Factors	Materials	Cycles	Results
	*D*	*L*_1_	*H*	*t*				
1	19	12	1.04	2.5	1.5	10	980,000	Broken
2	19	12	1.06	2.5	1.5	10	520,000	Broken
3	19	12	1.06	2.5	1.5	10	560,000	Broken
4	25	15	1.28	3.0	1.5	10	480,000	Broken
5	25	15	1.30	3.0	1.5	10	460,000	Broken
6	25	15	1.28	3.0	1.5	10	850,000	Broken
7	19	12	1.06	2	1.5	S30408	320,000	Broken
8	19	12	1.08	2	1.5	S30408	210,000	Broken
9	25	15	1.28	3	1.5	S30408	1,000,000	Unbroken
10	25	15	1.26	3	1.5	S30408	1,000,000	Unbroken

**Table 5:** Results of grid independence test for SG tube.

No. of Nodes (Million)	Stress Intensity (MPa)	Relative Change (%)
0.93	77.02	−1.17
2.8	77.53	−0.51
5.7	77.93	0

**Table 6:** Results of grid independence test for CD tube.

No. of Nodes (Million)	Stress Intensity (MPa)	Relative Change (%)
0.52	91.63	−3.05
1.2	91.81	−2.87
3.2	94.52	0

**Table 7:** Experimental verification of $K_{f}^{S}$ for the SG tubes.

No.	Tube Size				$K_{f}^{E}$	$K_{f}^{S}$	Error
	*D*	*P*	*ε*	*t*			
1	19	11.3	0.95	2	0.7583	0.6948	−8.37%
2	19	11	0.85	2	0.7668	0.7215	−5.91%
3	19	10.8	0.9	2	0.7625	0.6969	−8.60%
4	25	12.5	0.9	3.1	0.8521	0.8230	−3.42%
5	25	12.5	0.85	3	0.8724	0.8305	−4.80%
6	25	12.5	1	3	0.8293	0.7795	−6.01%

**Table 8:** Experimental verification of $K_{f}^{C}$ for the CD tubes.

No.	Tube Size				$K_{f}^{E}$	$K_{f}^{C}$	Error
	*D*	*L*_1_	*H*	*t*			
1	19	12	1.06	2.64	0.8481	0.7935	−6.44%
2	19	12	1.08	2.64	0.8252	0.7873	−4.59%
3	19	12	1.06	2.62	0.8367	0.7938	−5.12%
4	25	15	1.40	3	0.7983	0.7579	−5.06%
5	25	15	1.42	3	0.7896	0.7537	−4.55%
6	25	15	1.36	3	0.8463	0.7663	−9.45%

## Nomenclature

CD	Converging–diverging.	
*D*	Nominal outer diameter of the tube, mm.	
*F*	Axial loading, N.	
$F_{max}$	Maximum load, N.	
*H*	Rib height, mm.	
HEHE	Highly efficient heat exchange.	
$K_{1}^{}$	Axial elastic stiffness of the HEHE tubes, N/m.	
$K_{2}^{}$	Axial elastic stiffness of the smooth tubes, N/m.	
$K_{f}$	Stiffness equivalent factor.	
$K_{f}^{C}$	Stiffness equivalent factor of CD tubes.	
$K_{f}^{S}$	Stiffness equivalent factor of SG tubes.	
$K_{f}^{E}$	Stiffness equivalent factors obtained by experiments.	
*L*_1_	Length of a period section, mm.	
*L*_2_	Length of the tapering section, mm.	
*n*	Safety factor.	
*P*	Pitch of the tube, mm.	
*R*	Load ratio.	
$R_{Z}^{}$	Stress concentration factor.	
$R_{Z}^{C}$	Axial stress concentration factor of CD tubes.	
$R_{Z}^{S}$	Axial stress concentration factor of SG tubes.	
*S*	Cross-sectional area of the base tube, mm^2^.	
SG	Spirally grooved.	
*t*	Thickness of the tube, mm.	
*ε*	Depth of spiral groove, mm.	
$\sigma_{s}$	Yield strength of the material, MPa.	
$\sigma_{max}$	Maximum allowable stress, MPa.	
